# Clinical and Prognostic Significance of Tumor-Infiltrating CD8+ T Cells and PD-L1 Expression in Primary Gastrointestinal Stromal Tumors

**DOI:** 10.3389/fonc.2021.789915

**Published:** 2021-12-10

**Authors:** Xiangfei Sun, Ping Shu, Yong Fang, Wei Yuan, Qiang Zhang, Jianyi Sun, Min Fu, Anwei Xue, Xiaodong Gao, Kuntang Shen, Yingyong Hou, Yihong Sun, Jing Qin, Xinyu Qin

**Affiliations:** ^1^ Department of General Surgery, Zhongshan Hospital, Fudan University School of Medicine, Shanghai, China; ^2^ Department of Pathology, Zhongshan Hospital, Fudan University School of Medicine, Shanghai, China

**Keywords:** gastrointestinal stromal tumors, tumor-infiltrating lymphocyte, programmed cell death protein-1, PD-L1, programmed cell death protein-Ligand 1, prognosis

## Abstract

**Purpose:**

Immunotherapy for gastrointestinal stromal tumors (GISTs) remains a clinical challenge. The present study aimed to explore the clinical and prognostic significance of immune cell infiltration and PD-L1 expression in GISTs.

**Methods:**

A total of 507 clinical tissue specimens of primary GISTs were collected for immunohistochemical analysis of immune cell infiltration and PD-L1 expression. Influencing factors of survival were evaluated by Kaplan–Meier analysis. Univariate and multivariate analyses were performed using the Cox regression model.

**Results:**

There were significant differences in sex, tumor location, size, mitotic index, NIH risk grade, and cell morphology between different gene mutation types of GISTs. Immune cell infiltration in GISTs mainly involved macrophages and T cells. PD-1 was expressed in 48.5% of the tissue specimens, and PD-L1 expression was detected in 46.0% of the samples. PD-L1 expression was negatively correlated with the tumor size and mitotic index but positively correlated with the number of CD8+ T cells. There were significant differences in the number of CD8+ T cells between different gene mutation types. Wild type-mutant GISTs were enriched with CD8+ T cells as compared with KIT- and PDGFRA-mutant GISTs. The number of CD8+ T cells was higher in non-gastric GISTs. PD-L1 and CD8+ T cells were independent predictors for better relapse-free survival of GISTs.

**Conclusions:**

PD-L1 expression is a predictive biomarker for better prognosis of GISTs. Non-gastric GIST patients with wild-type mutations may be the beneficiaries of PD-1/PD-L1 inhibitors.

## Introduction

Gastrointestinal stromal tumors (GISTs) are the most common mesenchymal sarcomas of the gastrointestinal (GI) tract, accounting for 1–3% of all GI malignancies, with an incidence of 10–20 per million per year (1). Activating mutations of KIT have been identified in 80% GISTs, while platelet-derived growth factor receptor alpha (PDGFRA) mutations account for 5–10%. However, they may not be observed in the setting of KRAS mutation or SDH deficiency (2, 3). Imatinib mesylate (IM), a KIT tyrosine kinase inhibitor, has proved to be significantly beneficial in preventing recurrence and prolonging median survival by 1–5 years in patients with advanced and metastatic GISTs (4). However, about half of the patients will develop acquired resistance about one year after treatment. In addition, PDGFRA D842V and Wild-type (WT) GISTs are poorly responsive to IM. Although sunitinib, regorafenib, and ripretinib have been approved as the second-, third-, and fourth-line drugs, they can only prolong the progression-free survival (PFS) by 5.6, 4.8, and 6.3 months, respectively (5, 6). It is urgently necessary to develop more effective novel therapies for advanced GISTs.

Immunotherapy is now a standard treatment for many malignancies. The microenvironment of GISTs consists of tumor-infiltrating immune cells, which play a crucial role in tumor surveillance (7). Immune checkpoint inhibitors (ICIs) are a group of drug-targeting immune checkpoints. Programmed cell death-1(PD-1)/programmed cell death-ligand 1 (PD-L1) inhibitors have shown a lasting anti-tumor response and can improve the survival rate in several malignant tumors including kidney cancer, bladder cancer, melanoma, and lung cancer (8). In addition, the therapeutic efficacy of IM has been increased by combination with PD-1/PD-L1 inhibitors (9). However, only a few studies have focused on the immunotherapy of GISTs, and the role of PD-1/PD-L1 inhibitors in the treatment of GISTs is unclear. The present study aimed to explore the expression of PD-L1 in what we believe is one of the largest cohorts of primary untreated GISTs in an attempt to provide a basis for prognosis assessment and identify the beneficiaries of PD-1/PD-L1 inhibitors for GISTs.

## Materials and Methods

### Patient Selection

Included in this study were 507 patients with primary GISTs who underwent radical resection at the Zhongshan Hospital of Fudan University (Shanghai, China) between January 2013 and January 2020. None of them had been treated with immunotherapy, radiotherapy, or chemotherapy before surgery. Very low-risk and low-risk patients did not receive any postoperative therapy. Moderate-risk patients received IM adjuvant therapy for at least 1 year and high-risk patients for at least 3 years. Their clinicopathological features were retrieved from the medical records. The method of follow-up has been described in our previous article (10). Ethical approval was obtained from the ethics committee of the Zhongshan Hospital. All patients signed the informed consent and agreed to use their clinicopathological data and surgical specimens for scientific research.

### Immunohistochemistry

Representative GIST regions were marked on specific paraffin blocks according to the results of HE staining. Altogether 507 paraffin-embedded GIST tissue blocks were collected, and samples (2 mm × 6 mm) were acquired by inserting tissue array needles and then aligned on blank paraffin blocks to make a tissue microarray (TMA) (11). IHC was performed on 5-micron slices TMA on a fully automated immunohistochemistry machine (Leica Bond-Max), with the following antibodies: CD3, CD4, FoxP3, CD20, CD56, CD68, PD-1, and PD-L1. The complete list of the antibodies is provided in **Supplementary Table 1**. A fully automatic digital slice scanning system (Leica Aperio AT2) was used to scan the IHC staining images of each TMA. The tumor-infiltrating immune cells were counted in five high-power fields. PD-1 expression was evaluated by cell counting method, and PD-L1 expression by estimating the proportion of tumor cells (tumor proportion score, TPS), as negative when TPS was <1%, and as positive when TPS was ≥1%. All staining results were evaluated by two experienced pathologists (WY and YH) independently without knowledge of the clinical information. The detailed methods have been described in our previous article (10).

### Statistical Analysis

Statistical analyses were performed using SPSS statistical software version 26.0 and statistical figures were drawn by GraphPad Prism 8.0.2. According to the median number of immune cells, all GIST patients were divided into a low-expression group and a high-expression group. Continuous variable data are expressed as means ± standard deviation (SD). The correlation between the categorical variables was evaluated by Person’s chi-square test, continuity correction, or Fisher’s exact test depending on the specific situation. Kaplan–Meier analyses were used to evaluate the influencing factors of survival. Cox regression model was used for univariate and multivariate analysis. The statistically significant factors in univariate analysis were included in multivariate analysis. NIH risk classification is excluded because it is a combination of tumor size and mitotic index. All statistical tests were two-tailed at the 5% level of significance.

## Results

### Baseline Clinicopathological Data of the Patients

The clinicopathological data of the 507 GIST patients were collected in this study. The mean age was 59 ± 12 years, and the mean tumor size was 5.6 ± 3.8 cm. Of the 507 GISTs, 337 (66.5%) originated from the stomach, 60 (11.8%) from the duodenum, 93 (18.3%) from the small intestine, 7(1.4%) from the rectum, 4 (0.8%) from the esophagus, and 6 (1.2%) from other sites. The positive rates of CD117, CD34, and DOG-1 was 98.8% (501/507), 95.3% (483/507), and 98.8% (501/507), respectively. According to the modified NIH risk classification standard (12), there were 39 cases of very low risk (7.7%), 201 cases of low risk (39.6%), 107 cases of medium risk (21.1%), and 160 cases of high risk (31.6%). Histologically, 419 cases (82.6%) belonged to the spindle type, 25 (4.9%) to the epithelial type, and 63 (12.4%) to the mixed type **(** **Supplementary Table 2** **)**.

### Relationship Between Gene Mutation Types and Clinicopathological Features

In this study, there were 417 cases of KIT-mutant and 42 cases of PDGFRA-mutant GISTs. In addition, there are 48 patients with WT GISTs **(** **Supplement Table 3** **).** Statistical analysis showed that there were significant differences in gender (P = 0.037), tumor location (P <0.001), tumor size (P = 0.018), mitotic index (P = 0.001), risk grade (P <0.001), and cell morphology (P <0.001) between different gene mutation types. Patients with KIT mutated GISTs tended to have a larger tumor size and a higher mitotic index and risk grade as compared with those with PDGFRA-mutant and WT GISTs **(** **Table 1** **)**. There was no significant difference in tumor size, mitotic index, and risk grade between the KIT exon 9, 11, 13, and 17 mutated subgroups.

**Table 1:** Relationship between gene mutation types and clinicopathological features.

Factors	KIT (n = 417)	PDGFRA (n = 42)	Wild (n = 48)	*P*-value
Sex				**0.037**
male	179 (42.9%)	11 (26.2%)	23 (47.9%)	
female	238 (57.1%)	31 (73.8%)	25 (52.1%)	
Age (years)				0.340
≤60	214 (51.3%)	22 (52.4%)	30 (62.5%)	
>60	203 (48.7%)	20 (47.6%)	18 (37.5%)	
location				**<0.001**
Gastric	270 (64.7%)	42 (100.0%)	25 (52.1%)	
Non-Gastric	147 (35.3%)	0 (0.0%)	23 (47.9%)	
Tumor size				**0.018**
≤5 cm	235 (56.4%)	29 (69.0%)	36 (75.0%)	
>5 cm	182 (43.6%)	13 (31.0%)	12 (25.0%)	
Mitotic index				**0.001**
≤5/50HPF	279 (66.9%)	38 (90.5%)	40 (83.3%)	
>5/50HPF	138 (33.1%)	4 (9.5%)	8 (16.7%)	
NIH risk grade				**<0.001**
Very low-low	179 (42.9%)	28 (66.7%)	33 (68.8%)	
Moderate-high	238 (57.1%)	14 (33.3%)	15 (31.2%)	
Morphology				**<0.001**
Spindle	374 (89.7%)	15 (35.7%)	30 (62.5%)	
Epithelioid and Mixed	43 (10.3%)	27 (64.3%)	18 (37.5%)	

HPF, high-power fields; NIH, National Institutes of Health. All of our variables with p values less than 0.05 are in bold.

### Evaluation of Immune Cell Infiltration and PD-1/PD-L1 Expression

**Figure 1:**
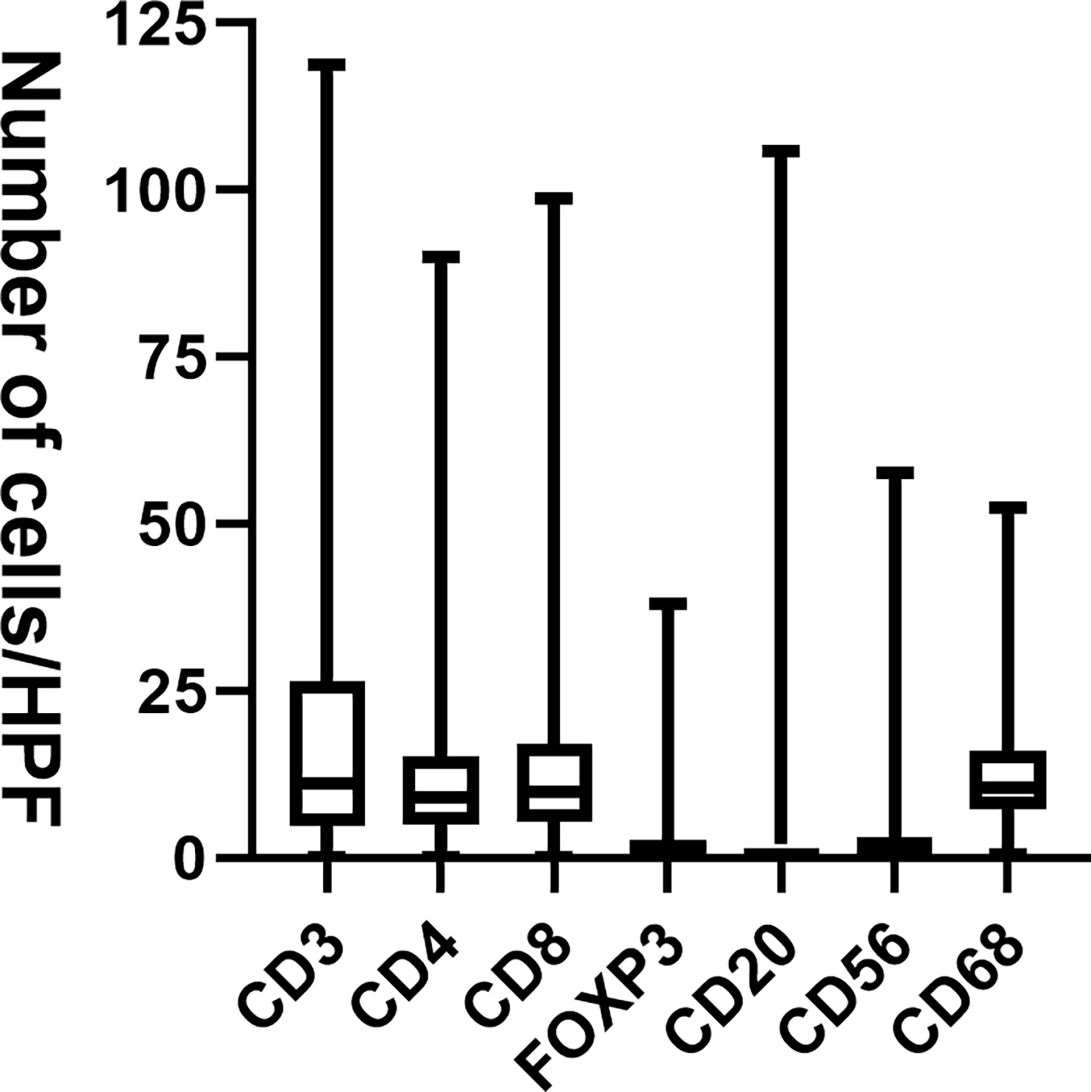
The number of tumor-infiltrating immune cells in GISTs. HPF, high power field.

In line with our previous study (11), immune cell infiltration mainly involved CD3+, CD8+, CD68+, and CD4+ in GISTs, and a small number of CD20+, CD56+, and Foxp3+ cells (**Figure 1**). Except that the nuclei of Foxp3 staining positive cells were reddish-brown, the other immunohistochemical staining positive cells were brown in the membrane (**Supplementary Figure 1**). PD-1 was expressed in 48.5% of the 507 GIST tissue specimens, and PD-L1 expression was detected in 46.0% of the samples. IHC analysis showed that the reddish-brown PD-1/PD-L1 positive signals were localized in the cell membrane of the GIST tissues (**Supplementary Figure 2**).

### CD8+ T Cells Are Preferentially Enriched in Non-Gastric GISTs With High PD-1/PD-L1 Expression

Univariate analysis showed that tumor-infiltrating CD8+ T cells were correlated with tumor location (P <0.001), size (P = 0.012), mitotic index (P = 0.001), risk grade (P = 0.004), gene mutation type (P <0.001), CD3+ (P <0.001), CD4+ (P <0.001), Foxp3+ T cells (P <0.001), CD20+ B cells (P <0.001), CD56+ NK cells (P <0.001), CD68+ macrophages (P = 0.003), PD-1 (P <0.001), and PD-L1 expression (P <0.001) **(** **Table 2** **)**. Multivariate analysis showed that the number of CD8+ T cells was positively correlated with CD3+ (P <0.001), CD4+ T cells (P <0.001), PD-1 (P <0.001), and PD-L1 (P = 0.038) expression. In addition, the number of CD8+ T cells in non-gastric GISTs was higher than that in gastric GISTs (P <0.001), and was higher in GISTs with size ≤5 cm than that in GISTs with size >5 cm (P = 0.022) (**Table 2**).

**Table 2:** Relationship between CD8+ T cells and clinicopathological features.

Factors	CD8+ T cell		P value	CD8+ T cell (low vs high)	
	Low	High		OR (95% CI)	P-value
Sex					
Female	110	103	0.890	–	–
Male	150	144			
Age(years)					
≤60	133	133	0.544	–	–
>60	127	114			
Location					
Gastric	192	145	**<0.001**	1	**<0.001**
Non-Gastric	68	102		2.935 (1.738–4.955)	
Tumor size					
≤5 cm	140	160	**0.012**	1	**0.022**
>5 cm	120	87		0.552 (0.332–0.919)	
Mitotic index					
≤5/50HPF	166	191	**0.001**	1	0.589
>5/50HPF	94	56		0.856 (0.487–1.505)	
NIH risk grade					
Very low-low	107	133	**0.004**	–	–
Moderate-high	153	114			
Morphology					
Spindle	219	200	0.333	–	–
Epithelioid and Mixed	41	47			
Mutation type					
KIT	232	185	**<0.001**	1	**<0.001**
PDGFRA and Wild	28	62		4.336 (2.176–8.641)	
CD3+ T cell					
Low	187	69	**<0.001**	1	**<0.001**
High	73	178		2.715 (1.610–4.578)	
CD4+ T cell					
Low	187	70	**<0.001**	1	**<0.001**
High	73	177		3.363 (1.943–5.820)	
Foxp3+ T cell					
Low	171	91	**<0.001**	1	0.568
High	89	156		1.169 (0.683–2.001)	
CD20+ B cell					
Low	158	97	**<0.001**	1	0.911
High	102	150		0.972 (0.594–1.591)	
CD56+ NK cell					
Low	166	107	**<0.001**	1	0.572
High	94	140		1.162 (0.691–1.953)	
CD68+ macrophage					
Low	151	111	**0.003**	1	0.509
High	109	136		0.848 (0.520–1.383)	
PD-1					
Low	200	61	**<0.001**	1	**<0.001**
High	60	186		4.433 (2.630–7.473)	
PD-L1					
Low	180	94	**<0.001**	1	**0.038**
High	80	153		1.650 (1.027–2.651)	

HPF, high-power fields; NIH, National Institutes of Health; PD-L1, programmed cell death-ligand 1. All of our variables with p values less than 0.05 are in bold.

### CD8+ T Cells Are Preferentially Enriched in WT Mutated GISTs

**Figure 2:**
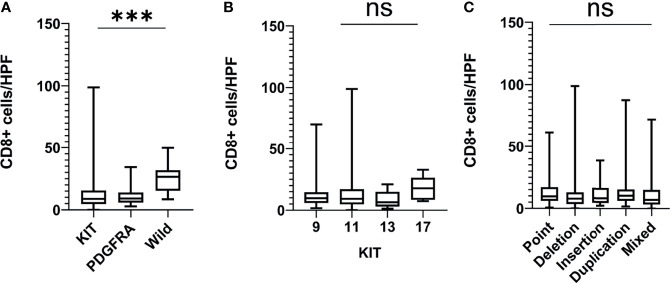
The number of CD8+ T cells in different gene mutation types of GISTs. **(A)** Wild-type mutated GISTs were enriched with CD8+ T cells as compared with KIT and PDGFRA mutated GISTs; **(B, C)** There was no significant difference in CD8+ T cell infiltration between the point mutation type, deletion mutation, insertion mutation, repeat mutation, and mixed mutation types or between the KIT exon 9, 11, 13, and 17 mutations. HPF, high-power field; ***P < 0.001; ns, no significance.

The further analysis showed that the number of CD8+ T cells in WT GISTs was higher than that in KIT and PDGFRA mutated GISTs (25.1 ± 10.3 *vs.* 12.6 ± 13.1 and 10.8 ± 6.9, both P <0.001). However, there was no significant difference in GISTs between point (13.0 ± 10.5), deletion (11.5 ± 14.0), insertion (12.0 ± 10.5), repetition (14.1 ± 15.1), and mixed (12.1 ± 13.5) mutations (P = 0.698) or between KIT exon 9 (12.3 ± 10.2), 11 (13.4 ± 13.2), 13 (8.8 ± 6.8), and 17 (17.4 ± 9.0) mutations (P = 0.292) **(** **Figure 2** **)**. In addition, our previous research demonstrated that there was also no statistical difference between PDGFRA exon 12 and 18 mutations (8.4 ± 4.8 *vs.* 11.5 ± 7.0, P = 0.350) (11).

### PD-L1 Expression Is Positively Correlated With CD8+ T Cells and PD-1 Expression, and Negatively Correlated With Tumor Size and Mitotic Index

We analyzed the correlations between PD-L1 expression and clinicopathological features in the 507 GIST patients, and found that PD-1 expression was correlated with tumor location (P = 0.048), mitotic index (P = 0.007), CD3+ (P <0.001), CD4+ (P <0.001), CD8+ (P <0.001), Foxp3+ T cell (P <0.001), CD20+ B cell (P <0.001), CD56+ NK cell (P = 0.006), CD68+ macrophages (P <0.001), and PD-L1 (P <0.001). The expression of PD-L1 was correlated with tumor size (P = 0.001), mitotic index (P <0.001), risk grade (P <0.001), CD3+ T cells (P <0.001), CD4+ T cells (P <0.001), CD8+ T cells (P <0.001), Foxp3+ T cells (P <0.001), CD20+ B cells (P = 0.007), CD56+ NK cells (P = 0.010), PD-1 (P <0.001), and CD68+ macrophages (P = 0.027) (**Supplementary Table 4**). Multivariate analysis showed that PD-1 expression was positively correlated with CD3+ (P <0.001), CD4+ (P <0.001), CD8+ T cells (P <0.001), Foxp3+ T cell (P <0.001), and CD20+ B cells (P = 0.001) and non-gastric GISTs (P = 0.020). The expression of PD-L1 was positively correlated with CD8+ T cells (P = 0.028) and PD-1 (P = 0.018), but negatively correlated with tumor size (P = 0.017) and mitotic index (P = 0.014) (**Table 3**).

**Table 3:** Multivariate analysis of the relationship between PD-1/PD-L1 expression and clinicopathological features.

Factors	PD-1 (negative vs positive)		PD-L1 (negative vs positive)	
	OR (95% CI)	P-value	OR (95% CI)	P-value
Location				
Gastric	1	**0.020**	–	–
Non-Gastric	1.891 (1.105–3.238)			
Tumor size				
≤5 cm	–	–	1 0.597 (0.391–0.912)	**0.017**
>5 cm				
Mitotic index				
≤5/50HPF >5/50HPF	1 0.617 (0.351–1.085)	0.094	1 0.560 (0.352–0.890)	**0.014**
CD3+ T cell				
Low	1	**<0.001**	1	0.061
High	2.544 (1.537–4.212)		1.574 (0.980–2.528)	
CD4+ T cell				
Low	1	**<0.001**	1	0.072
High	2.474 (1.488–4.112)		1.539 (0.961–2.463)	
CD8+ T cell				
Low	1	**<0.001**	1	**0.028**
High	4.226 (2.555–6.989)		1.669 (1.056–2.639)	
Foxp3+ T cell				
Low	1	**<0.001**	1	0.393
High	2.632 (1.569–4.416)		1.217 (0.775–1.912)	
CD20+ B cell				
Low	1	**0.001**	1	0.504
High	2.314 (1.415–3.785)		0.867 (0.570–1.319)	
CD56+ NK cell				
Low	1	0.530	1	0.445
High	0.854 (0.523–1.396)		1.167 (0.785–1.735)	
CD68+ macrophage				
Low	1	0.057	1	0.684
High	1.616 (0.986–2.648)		1.089 (0.724–1.638)	
PD-1				
Negative	–	–	1	**0.018**
Positive			1.812 (1.109–2.959)	
PD-L1		**0.036**		–
Negative	1		–	
Positive	1.686 (1.034–2.748)			

HPF, high-power fields; NIH, National Institutes of Health; PD-L1, programmed cell death-ligand 1. All of our variables with p values less than 0.05 are in bold.

### PD-L1 Expression and CD8+ T Cells Are Independent Prognostic Factors for GISTs

**Figure 3:**
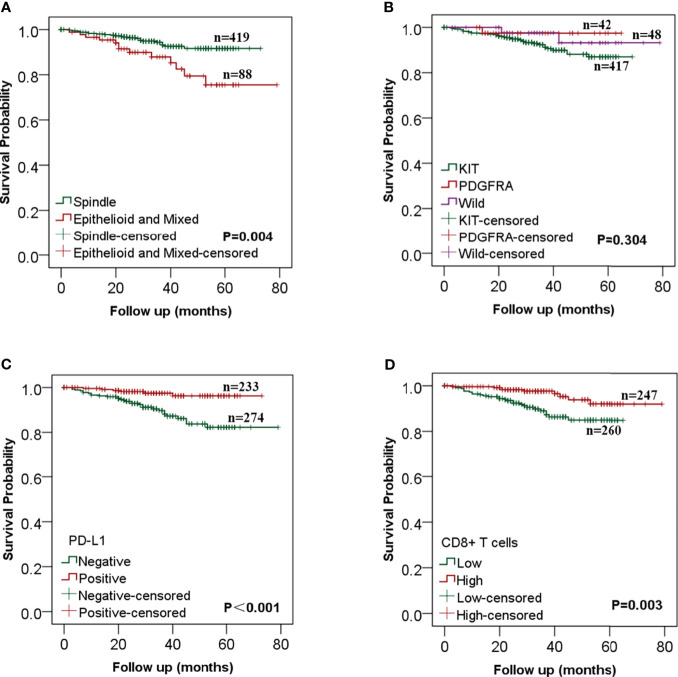
Kaplan–Meier analysis of the relationships between clinicopathological features and recurrence-free survival in gastrointestinal stromal tumors (GISTs). **(A)** GIST patients with the spindle cell type had a better RFS than those with epithelial and mixed cell; **(B)** There was no statistical difference in RFS between different mutation types; **(C)** RFS was significantly longer in patients with high PD-L1 expression than that in patients with low PD-L1 expression; **(D)** The increase of CD8+ T cells was significantly correlated with better RFS.

The survival and recurrence status was last updated in August 2020. Of the 507 included patients, 482 patients (95.1%) were followed up completely, and the rest 25 were lost to follow-up. The median follow-up period was 33 (0–79) months. By the time of the last follow-up, recurrence occurred in 36 cases (7.1%) and death in 4 (0.8%) cases. The overall 3- and 5-year RFS rates were 93.2 and 88.4% respectively. Log-rank analysis showed that the RFS was significantly longer in patients with tumor diameter ≤5 cm (P = 0.027), mitotic index ≤5/50HPF (P <0.001) (**Supplementary Figure 3**), spindle cell type (P = 0.004), high expression of PD-L1 (P <0.001), and high CD8+ T cell infiltration (P = 0.003) (**Figure 3**).

Univariate COX analysis showed that RFS was correlated with tumor size (P = 0.030), mitotic index (P <0.001), NIH risk grade (P = 0.007), cell type (P = 0.005), CD8+ T cells (P = 0.004) and PD-L1 expression (P = 0.024) (**Table 4**). A multivariate Cox regression model was used to analyze the factors affecting RFS. The results showed that mitotic index >5/50HPF (HR: 3.560, 95%CI: 1.700–7.454, P= 0.001), epithelial and mixed cell type (HR: 2.550, 95%CI: 1.263–5.149, P= 0.009) were independent risk factors for recurrence. The expression of PD-L1 (HR: 0.370, 95%CI: 0.150–0.911, P = 0.031) and CD8+ T cells (HR: 0.409, 95%CI: 0.185–0.900, P = 0.026) were independent protective factors affecting RFS of GIST patients, but tumor size was not an independent risk factor (**Table 4**).

**Table 4:** Univariate and multivariate Cox regression analysis on variables affecting recurrence-free survival.

Factors	Univariate		Multivariate	
	HR (95% CI)	P-value	HR (95% CI)	P-value
Sex				
Female	1	0.756	–	–
Male	1.112 (0.569–2.174)			
Age(years)			–	–
≤60	1	0.124		
>60	1.693 (0.866–3.309)			
Location			–	–
Gastric	1	0.800		
Non-Gastric	1.091 (0.557–2.135)			
Tumor size				
≤5 cm	1	**0.030**	1	0.879
>5 cm	2.079 (1.072–4.034)		1.057 (0.521–2.143)	
Mitotic index				
≤5/50HPF	1	**<0.001**	1	**0.001**
>5/50HPF	4.991 (2.496–9.982)		3.560 (1.700–7.454)	
NIH risk grade			–	–
Very low-low	1	**0.007**		
Moderate-high	2.802 (1.318–5.959)			
Morphology				
Spindle	1	**0.005**	1	**0.009**
Epithelioid and Mixed	2.638 (1.335–5.212)		2.550 (1.263–5.149)	
Mutation type			–	–
KIT	1	0.364		
PDGFRA and Wild	1.810 (0.364–8.998)			
CD3+ T cell			–	–
Low	1	0.325		
High	0.714 (0.365–1.397)			
CD4+ T cell			–	–
Low	1	0.315		
High	0.709 (0.362–1.387)			
CD8+ T cell				
Low	1	**0.004**	1	**0.026**
High	0.331 (0.156–0.705)		0.409 (0.185–0.900)	
Foxp3+ T cell			–	–
Low	1	0.861		
High	0.943 (0.488–1.822)			
CD20+ B cell			–	–
Low	1	0.184		
High	0.635 (0.325–1.241)			
CD56+ NK cell			–	–
Low	1	0.392		
High	0.746 (0.382–1.459)			
CD68+ macrophage			–	–
Low	1	0.923		
High	1.033 (0.537–1.998)			
PD-1			–	–
Negative	1	0.211		
Positive	0.648 (0.328–1.280)			
PD-L1				
Negative	1	**0.024**	1	**0.031**
Positive	0.433 (0.209–0.898)		0.370 (0.150–0.911)	

HPF, high-power fields; NIH, National Institutes of Health; PD-L1, programmed cell death-ligand 1. All of our variables with p values less than 0.05 are in bold.

## Discussion

Gastrointestinal stromal tumors are the most common mesenchymal malignancies of the GI tract, with an annual incidence of about 10–20 per million (1). Studies have shown that the gene mutation type is associated with tumor location, size, mitotic index, risk grade, and prognosis (13, 14). Our results showed that the type of gene mutation was not only related to these factors but showed significant differences in sex and cell types. In addition, patients with KIT mutated GISTs tended to have an increased tumor size and a higher mitotic index and risk grade as compared with those with PDGFRA and WT-mutated GISTs. Therefore, we believe that the malignant potential of KIT mutated GISTs is higher than that of other mutant types.

IM is the first-line drug for the treatment of recurrent, metastatic, and unresectable GISTs at present, knowing that it is a tyrosine kinase inhibitor that can significantly improve the prognosis of GIST patients. Although the tumor response to IM is impressive, resistance usually develops within 2 years due to secondary gene mutations (15). The efficacy of IM increment or replacement of second-, third-, or fourth-line drugs is very limited (5, 6). Therefore, more effective new drugs are needed for the treatment of GISTs, especially in patients who have developed drug resistance. The tumor immune microenvironment plays an important role in tumor growth and progression (16). Immunotherapies are potential therapeutic agents for GISTs. They are currently not the standard of care, but some studies have shown that there is a higher proportion of macrophages and T cells, and a lower proportion of B cells, DC, and NK cells in GISTs (17). Our results showed that there was mainly immune cell infiltration of CD3+, CD8+, CD68+, and CD4+ in GISTs, with a small number of CD20+, CD56+, and Foxp3+ cells. Previous studies have also demonstrated that tumor-infiltrating immune cells are not only related to the clinicopathological features but also associated with the prognosis of GIST patients. Some studies reported that infiltrating macrophage was the mainly M2 type, and the number of macrophages in metastatic GISTs was twice as much as that in primary GISTs (18). The number of CD68+ macrophages was negatively correlated with tumor size and metastasis but positively correlated with tumor recurrence risk and prognosis (19). Cameron et al. found that the number of CD20+ B and CD3+ T cells in the metastatic tumor was higher than that in the primary tumor (20). However, few studies have addressed tumor-infiltrating CD8+ T cells in GISTs. Our results showed that the number of CD8+ T cells in non-gastric GISTs was higher than that in gastric GISTs, and was higher in GISTs with size ≤5 cm than that in GISTs with size >5 cm. Besides, survival analysis also showed that CD8+ T cell infiltration was an independent protective factor affecting RFS of GIST patients. High CD8+ T cell infiltration was associated with improved RFS.

Immune checkpoints refer to a series of molecules expressed on immune cells which play an important role in preventing autoimmunity by regulating the degree of immune activation (21). Immune escape occurs when the expression of immune checkpoint-related ligands is upregulated in malignant tumors. The most successful immune checkpoint blockade therapy is anti-PD-1/PD-L1 therapy. PD-L1 expressed in malignant tumors binds to PD-1 expressed in activated T cells, thus weakening the CD8+T cell proliferation and inhibiting the T cell receptor signal pathway. PD-1/PD-L1 inhibitors can inhibit this process and have been approved to treat a wide variety of cancer types including lung cancer, kidney cancer, bladder cancer, and melanoma (22–24). Nowadays, Pembrolizumab, Nivolumab, and Atezolizumab have shown definite efficacy and can improve the prognosis (25, 26).

It is very important to find appropriate predictive parameters that can indicate the efficacy of PD-1/PD-L1 inhibitors in GISTs. One study compared several predictive parameters for the use of PD-1/PD-L1 inhibitors in non-small cell lung cancer (NSCLC) and believed that the expression of PD-1/PD-L1 was the most valuable predictor (27). Some other studies found that PD-1 was highly expressed in infiltrating T cells of GISTs, while the expression of PD-L1 was highly heterogeneous (9). One study analyzed the mRNA expression data of 139 cases of primary GISTs and found that the expression of PD-L1 was heterogeneous among GISTs and was higher in the samples classified as low risk by the American Institute of military Pathology (AFIP) (28). In addition, Pantaleo et al. found that there was co-expression of PD-L1 and CD8+ T cells in GISTs, and IM could downregulate the expression of PD-L1 by inhibiting KIT and PDGFRA to counteract the immunosuppression of GISTs (29). Zhao et al. found that PD-1/PD-L1 blocking could reduce the apoptosis of CD8+ T cells in GISTs by regulating the PI3K/Akt/mTOR signaling pathway (30). However, few studies have focused on the prognosis of PD-1/PD-L1. Our results showed that the rate of PD-L1 expression was 46.0% and was negatively correlated with tumor size and mitotic index. In addition, PD-L1 expression was an independent protective factor for RFS. Most studies have shown that the high expression of PD-L1 is associated with poor prognosis because PD-L1 negatively regulated the anti-tumor response of T cells (31, 32). On the contrary, in some malignant tumors, the high expression of PD-L1 was found to be associated with a good prognosis (33). It was found in our study that high expression of PD-L1 was associated with the increase of CD8+ T cells, based on which we speculate that PD-L1 expression is induced by CD8+ T cell infiltration, and upregulation of PD-L1 expression is due to the feedback of inhibition of the anti-tumor immune activity in GISTs. All these results suggest that PD-L1 expression may inhibit tumor growth and could be used as an independent tumor marker of GISTs to predict the risk of recurrence and the efficacy of PD-1/PD-L1 inhibitors.

All the above preclinical studies indicate that PD-1/PD-L1 inhibitors have a broad application prospect in GISTs. However, there have been few clinical trials with PD-1/PD-L1 inhibitors and currently available clinical data are not very promising. A multi-center phase II clinical trial on PD-1 inhibitors combined with regular cyclophosphamide chemotherapy in 50 patients with advanced sarcoma showed that only 10 patients with advanced GISTs escaped disease progression at 6 months. They believed that the limited efficacy may be related to the immunosuppressive tumor microenvironment due to the activation of tumor-associated macrophages (TAMs) and indoleamine-2,3-dioxygenase (IDO) pathway (34). However, we believe that the unsatisfactory efficacy may also be related to the small sample size and the choice of beneficiaries. In addition to the PD-1/PD-L1 expression, driver genes are also a very important factor affecting the efficacy of immunotherapy. Driver genes refer to genes that are closely related to tumorigenesis. It was reported that the expression of PD-L1 in TP53 or KRAS mutant NSCLC was higher than that in WT NSCLC (35). A phase II clinical trial using the PD-L1 inhibitor Durvalumab as third-line therapy for advanced NSCLC showed that the drug in NSCLC with EGFR−/ALK− was more effective than that in NACLC with EGFR+/ALK+ regardless of PD-L1 expression (36). A clinical study using IM in combination with the CTLA-4 inhibitor Ipilimumab in 10 patients with advanced GISTs showed that 9 patients (8 with mutations in KIT exon 11 and 1 with a mutation in KIT exon 13) all progressed, but in 1 case of WT GIST, the tumor shrank by 68% (37). Our results showed that the number of CD8+ T cells in WT GISTs was significantly higher than that in KIT and PDGFRA mutant GISTs, and PD-L1 was positively correlated with CD8+ T cells, suggesting that WT GISTs may benefit from PD-1/PD-L1 inhibitors.

This study has certain limitations. Further research is needed to elucidate the mechanisms of heterogeneous PD-1/PD-L1 expression. In addition, the findings and conclusions of this study need to be verified in larger-sample clinical trials to ensure the safety and efficacy of PD-1/PD-L1 inhibitors in GISTs before they can be applied to clinical practice.

## Conclusion

We investigated the tumor-infiltrating immune cells and PD-1/PD-L1 expression in GISTs and analyzed the correlation between the clinicopathological characteristics of GISTs and the prognosis. Many tumor-infiltrating CD8+ T cells and heterogeneous expression of PD-1/PD-L1 were detected in GISTs. In addition, the expression of PD-L1 was negatively correlated with tumor size and mitotic index, and the number of CD8+ T cells was positively correlated with the expression of PD-L1 and was higher in non-gastric GISTs with WT mutations. CD8+ T cells and PD-L1 expression were independent protective factors for a better outcome, suggesting that PD-1/PD-L1 inhibitors may prove to be a promising strategy for the treatment of GISTs, and non-gastric GISTs patients with WT mutations may be the beneficiaries.

## Data Availability Statement

The original contributions presented in the study are included in the article/**Supplementary Material**. Further inquiries can be directed to the corresponding authors.

## Ethics Statement

Written informed consent was obtained from the individual(s) for the publication of any potentially identifiable images or data included in this article.

## Author Contributions

XS and PS designed the work and wrote the manuscript. QZ, JS, AX, and MF analyzed and interpreted the patient data. WY and YH performed the immunohistochemistry examinations. YF, XG, and KS revised the manuscript. YS, JQ, and XQ were responsible for the management and coordination of the planning and execution of research activities XS, PS, and YF were major contributors in writing the manuscript. All authors contributed to the article and approved the submitted version.

## Funding

This study was supported by the National Natural Science Foundation of China (81773080).

## Conflict of Interest

The authors declare that the research was conducted in the absence of any commercial or financial relationships that could be construed as a potential conflict of interest.

## Publisher’s Note

All claims expressed in this article are solely those of the authors and do not necessarily represent those of their affiliated organizations, or those of the publisher, the editors and the reviewers. Any product that may be evaluated in this article, or claim that may be made by its manufacturer, is not guaranteed or endorsed by the publisher.

## Abbreviations

GISTs, Gastrointestinal stromal tumors; PDGFRA, Platelet-derived growth factor receptor alpha; WT, Wild-type; HPF, High-power field; ICIs, Immune checkpoint inhibitors; IM, Imatinib Mesylate; SU, Sunitinib Malate; TKI, Tyrosine kinase inhibitor; OS, Overall survival; RFS, Relapse free survival; PD-1, Programmed cell death protein-1; PD-L1, Programmed cell death protein-Ligand 1; TIL, Tumor-infiltrating lymphocyte; Treg, Regulatory T cell; CD, Cluster of differentiation; AFIP, Armed Forces Institute of pathology; NCCN, National Comprehensive Cancer Network; NIH, National Institutes of Health; NSCLC, Non-small-cell lung cancer; HE, Hematoxylin and eosin stain; TMA, Tissue microarray; 95%CI, 95% confidence interval; HR, Hazard ratio; NS, Not significant.
