# Supplementary material for: Clinical and Prognostic Significance of Tumor-Infiltrating CD8+ T Cells and PD-L1 Expression in Primary Gastrointestinal Stromal Tumors
Source: Front Oncol. 2021 Dec 10;11:789915. doi: 10.3389/fonc.2021.789915 (PMC8709532; doi:10.3389/fonc.2021.789915)
Supplement: Supplementary file 1 [file DataSheet_1.docx]

**Supplementary Table 1. Antibodies used in immunohistochemical staining**

|  | Antibody | Clone | Company |
| --- | --- | --- | --- |
| T cells | mouse CD3 McAb | LN10 | Leica |
| Th cells | mouse CD4 McAb | 4B12 | DAKO |
| Treg cells | mouse Foxp3 McAb | 236A1E7 | Abcam |
| B cells | mouse CD20 McAb | L26 | DAKO |
| NK cells | mouse CD56 McAb | 1B6 | Leica |
| Macrophages | mouse CD68 McAb | PGM1 | DAKO |
| CTL | rabbit CD8 PcAb | POLY | Abcam |
| PD-1 | rabbit PD-1 McAb | EPR4877 | Abcam |
| PD-L1 | rabbit PD-L1 McAb | EIL3N | CST |

**Supplementary Table 2. Clinicopathological characteristics of the 507 GIST patients**

| Factors | Total (n=507) |
| --- | --- |
| Sex |  |
| male | 213(42.0%) |
| female | 294(58.0%) |
| Age(years) |  |
| ≤ 60 | 266(52.5%) |
| > 60 | 241(47.5%) |
| location |  |
| Gastric | 337(66.5%) |
| Non-Gastric | 170(33.5%) |
| Tumor size |  |
| ≤ 5cm | 300(59.2%) |
| > 5cm | 207(40.8%) |
| Mitotic index |  |
| ≤5/50HPF | 357(70.4%) |
| >5/50HPF | 150(29.6%) |
| NIH risk grade |  |
| Very low-low | 240(47.3%) |
| Moderate-high | 267(52.7%) |
| Morphology |  |
| Spindle | 419(82.6%) |
| Epithelioid and Mixed | 88(17.4%) |

HPF: high-power fields; NIH: National Institutes of Health

**Supplement Table 3. Gene mutation types in 507 GIST cases**

| Mutation types | Total |  | | | | |
| --- | --- | --- | --- | --- | --- | --- |
|  |  | Point | Deletion | Insertion | Duplication | Mixed |
| KIT | 417 |  |  |  |  |  |
| 9 | 30 | 0 | 0 | 1 | 29 | 0 |
| 11 | 374 | 134 | 161 | 14 | 20 | 45 |
| 13 | 8 | 7 | 1 | 0 | 0 | 0 |
| 17 | 5 | 5 | 0 | 0 | 0 | 0 |
| PDGFRA | 42 |  |  |  |  |  |
| 12 | 5 | 3 | 0 | 0 | 0 | 2 |
| 18 | 37 | 28 | 6 | 0 | 0 | 3 |
| Wild type | 48 | - | - | - | - | - |

**Supplement Table 4. Univariate analysis of the relationship between PD-1/PD-L1 expression and clinicopathologic features**

| Factors | PD-1 | |  | PD-L1 | |  |
| --- | --- | --- | --- | --- | --- | --- |
|  | Negative | Positive | P-value | Negative | Positive | P-value |
| Sex  Female  Male | 110(42.1%)  151(57.9%) | 103(41.9%)  143(58.1%) | 0.950 | 114(41.6%)  160(58.4%) | 99(42.5%)  134(57.5%) | 0.841 |
| Age(years)  ≤60  >60 | 138(52.9%)  123(47.1%) | 128(52.0%)  118(48.0%) | 0.850 | 135(49.3%)  139(50.7%) | 131(56.2%)  102(43.8%) | 0.118 |
| Location  Gastric  Non-Gastric | 184(70.5%)  77(29.5%) | 153(62.2%)  93(37.8%) | **0.048** | 190(69.3%)  84(30.7%) | 147(63.1%)  86(36.9%) | 0.137 |
| Tumor size  ≤5cm  ＞5cm | 156(59.8%)  105(40.2%) | 144(58.5%)  102(41.5%) | 0.778 | 144(52.6%)  130(47.4%) | 156(67.0%)  77(33.0%) | **0.001** |
| Mitotic index  ≤5/50HPF  ＞5/50HPF | 170(65.1%)  91(34.9%) | 187(76.0%)  59(24.0%) | **0.007** | 172(62.8%)  102(37.2%) | 185(79.4%)  48(20.6%) | **＜0.001** |
| NIH risk grade  Very low-low  Moderate-high | 120(46.0%)  141(54.0%) | 120(48.8%)  126(51.2%) | 0.527 | 109(39.8%)  165(60.2%) | 131(56.2%)  102(43.8%) | **＜0.001** |
| Morphology  Spindle  Epithelioid and Mixed | 220(84.3%)  41(15.7%) | 199(80.9%)  47(19.1%) | 0.313 | 227(82.8%)  47(17.2%) | 192(82.4%)  41(17.6%) | 0.896 |
| Mutation type  KIT  PDGFRA  Wild | 218(83.5%)  21(8.0%)  22(8.4%) | 199(80.9%)  21(8.5%)  26(10.6%) | 0.685 | 230(83.9%)  24(8.8%)  20(7.3%) | 187(80.3%)  18(7.7%)  28(12.0%) | 0.189 |
| CD3+ T cell  Low  High | 195(74.7%)  66(25.3%) | 61(24.8%)  185(75.2%) | **＜0.001** | 173(63.1%)  101(36.9%) | 83(35.6%)  150(64.4%) | **＜0.001** |
| CD4+ T cell  Low  High | 195(74.7%)  66(25.3%) | 62(25.2%)  184(74.8%) | **＜0.001** | 175(63.9%)  99(36.1%) | 82(35.2%)  151(64.8%) | **＜0.001** |
| CD8+ T cell  Low  High | 200(76.6%)  61(23.4%) | 60(24.4%)  186(75.6%) | **＜0.001** | 180(65.7%)  94(34.3%) | 80(34.3%)  153(65.7%) | **＜0.001** |
| Foxp3+ T cell  Low  High | 187(71.6%)  74(28.4%) | 75(30.5%)  171(69.5%) | **＜0.001** | 167(60.9%)  107(39.1%) | 95(40.8%)  138(59.2%) | **＜0.001** |
| CD20+ B cell  Low  High | 173(66.3%)  88(33.7%) | 82(33.3%)  164(66.7%) | **＜0.001** | 153(55.8%)  121(44.2%) | 102(43.8%)  131(56.2%) | **0.007** |
| CD56+ NK cell  Low  High | 156(59.8%)  105(40.2%) | 117(47.6%)  129(52.4%) | **0.006** | 162(59.1%)  112(40.9%) | 111(47.6%)  122(52.4%) | **0.010** |
| CD68+ macrophage  Low  High | 163(62.5%)  98(37.5%) | 99(40.2%)  147(59.8%) | **＜0.001** | 154(56.2%)  120(43.8%) | 108(46.4%)  125(53.6%) | **0.027** |
| PD-1  Negative  Positive | - | - | - | 181(66.1%)  93(33.9%) | 80(34.3%)  153(65.7%) | **＜0.001** |
| PD-L1  Negative  Positive | 181(69.3%)  80(30.7%) | 93(37.8%)  153(62.2%) | **＜0.001** | - | - | - |

HPF: high-power fields; NIH: National Institutes of Health; PD-L1: programmed cell death-ligand 1


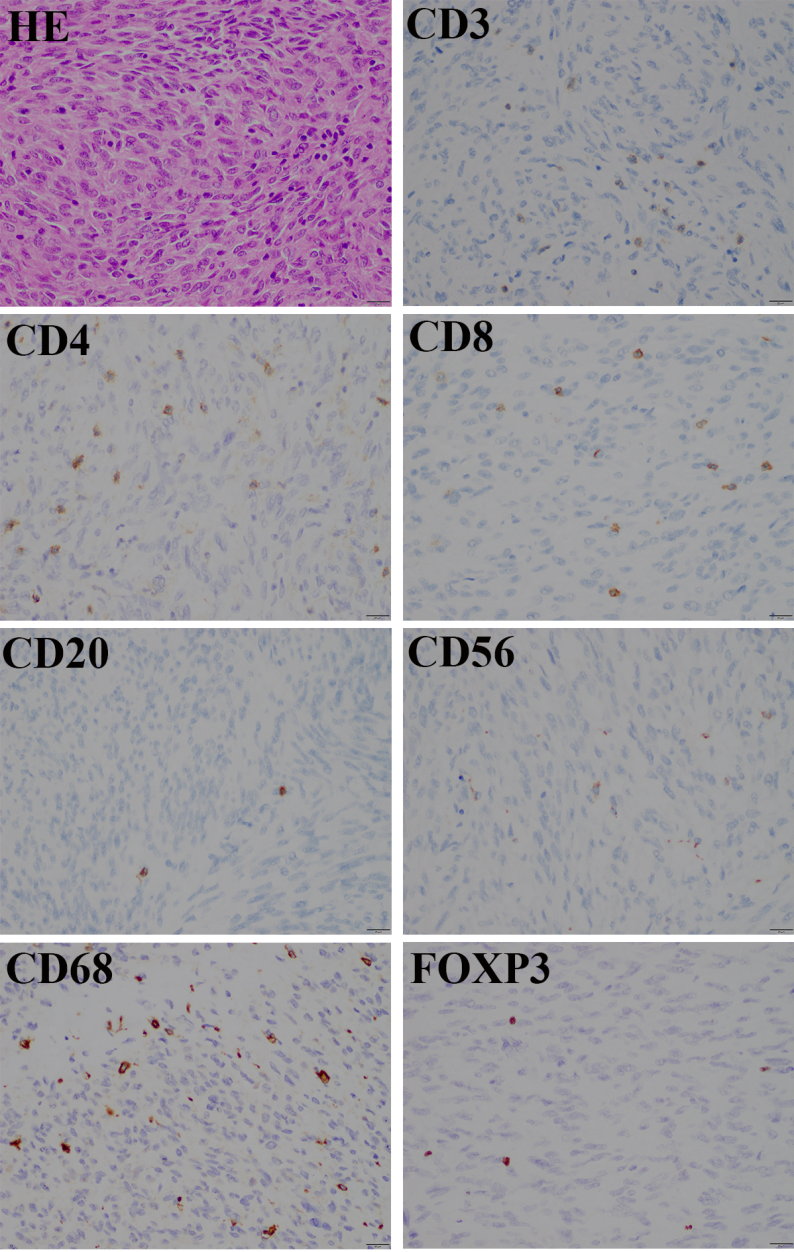


**Supplement Fig. 1** **Representative immunohistochemical photos of tumor-infiltrating immune cells in gastrointestinal stromal tumors(X 400).**

HE, hematoxylin and eosin staining


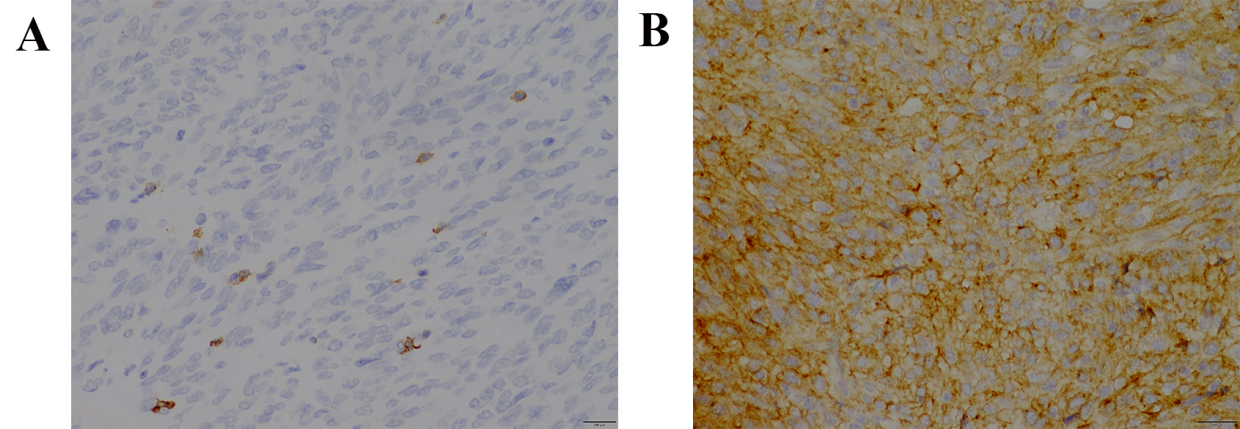


**Supplement Fig. 2** **Representative immunohistochemical staining for PD-1/PD-L1 in gastrointestinal stromal tumors (X 400).**

A: High cytoplasmic staining of PD-1 in the cytoplasm; B: Specific high positive staining for PD-L1 in the tissue microarray samples.


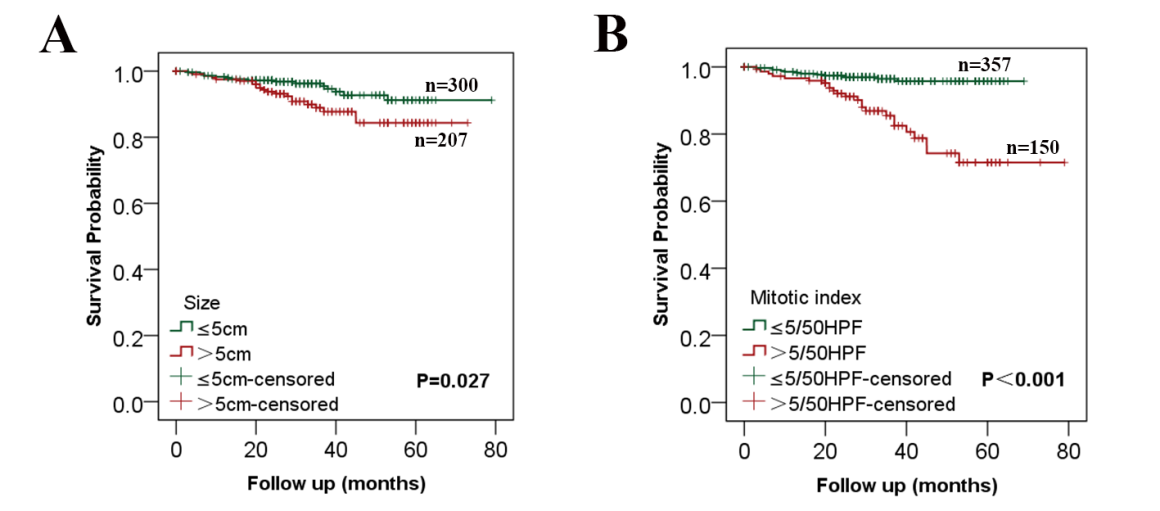


**Supplement Fig. 3 Kaplan-Meier analysis of the relationships between clinicopathological features and recurrence-free survival in gastrointestinal stromal tumors (GISTs).**

A: Patients with tumor diameter ≤ 5 cm had a better RFS than patients with diameter > 5 cm; B: RFS was significantly longer in patients with mitotic index ≤ 5/50HPF than that in patients with mitotic index > 5/50HPF;
